# 

**Published:** 1906-02-17

**Authors:** 


### NOTICE TO CORRESPONDENTS.

All MSS., Letters, Books for Review, and other matters intended for the Editor should be addressed THE EDITOR, "THE HOSPITAL," THE HOSPITAL BUILDINGS, 28 & 29 SOUTHAMPTON STREET, STRAND, W.C.

The Editor cannot undertake to return rejected MS., even when accompanied by stamped directed envelope.

**Notice to Advertisers and Subscribers.**

All Advertisements, Orders for Copies of THE HOSPITAL, and Business Communications should be addressed to **THE MANAGER (Not to the Editor),** THE HOSPITAL, 28 & 29 Southampton Street, Strand London, W.C.

The Cost of Subscription, post free, is as follows:—Per week, 3½d.; per quarter, 3s. 3d.; per half-year, 6s. 6d.; per annum, 13s. Foreign and Colonial:—Per annum, 19s. 6d., payable, in all cases, in advance.

**NOTICE.—Vol. XXXVIII. of "The Hospital" April to September 1905), in handsome cloth binding, will shortly be on sale at the office of this Journal, price 10s. 6d. Cases for binding the Numbers contained in this Volume 2s. Index to Vol. XXXVIII., 3½d., post free.**

**The Monthly Part for February is now ready. Price 1s., by post, 1s. 3d.**

### BOOKS RECEIVED.

Baillière, Tindall, and Cox.

"The 'Nauheim' Treatment of Chronic Diseases of the Heart." 2nd edition. By Leslie Thorne Thorne, M.D.

J. Bale, Sons and Danielsson, Limited.

"Patent Foods and Patent Medicines." By Robert Hutchison, M.D.

J. and A. Churchill.

"A Preliminary Inquiry Concerning the Milk Supply of Schools." By C. E. Shelly, M.D.

H. J. Glaisher.

"Oliver Wendell Holmes and Puerperal Fever." By Chas. J. Cullingworth, M.D.

Hazell, Watson and Viney, Limited.

"The Game of Ju-Jitsu." By Taro Miyake and Yukio Tani.

Whittaker and Co.

"Radiography." By S. R. Bottone.

The Scientific Press, Limited.

"The Nursing of Infectious Diseases." By F. J. Woollacott.

### MEDICAL APPOINTMENTS AND VACANCIES.

### VACANCIES.

Bury Infirmary.—Senior House Surgeon.
City of London Hospital for Diseases of the Chest, Victoria Park, E.—Two Physicians to Out-patients.
Denbigh, Denbighshire Infirmary.—House Surgeon.
Hospital for Sick Children, Great Ormond Street, London, W.C.—Asistant Surgeon.
Hull Royal Infirmary.—House Surgeon.
Ingham Infirmary and South Shields and Westoe Dispensary.—Assistant House Surgeon.
Larbert, Stirling District Asylum.—Senior Assistant Medical Officer.
Leicester Infirmary.—House Surgeon.
Lincoln County Hospital.—Senior House Surgeon, unmarried.
Manchester, St. Mary's Hospital, Clifford Street.—House Surgeon.
Metropolitan Hospital, Kingsland Road, N.E.—Assistant Physician.
New Hospital for Women, Euston Road.—House Surgeon. Also Non-Resident House Surgeon.
Torbay Hospital, Torquay.—House Surgeon.
Winchester, Royal Hants County Hospital.—House Physician.

### TO ATTAIN SUCCESS IN NURSING

KNOWLEDGE IS THE ROOT OF SUCCESS

as in any other profession, it is essential that you should obtain the best teachers.

This is possible by procuring and studying the works of those who have themselves attained success.

The undermentioned books are all written by experts in the subjects of which they treat, and will therefore prove of the utmost assistance to you in acquiring the knowledge that is so necessary if you are to succeed in the fierce competition of to-day.

SUCCESS IS THE FRUIT OF KNOWLEDGE

**ART OF MASSAGE.**
By Mrs. Creighton Hale.
Illustrated, 6/- post free.

**MEDICAL ELECTRICITY AND LIGHT TREATMENT.**
By Sister Kate Neale.
Illustrated, 2/6 net, 2/9 post free.

**OPHTHALMIC NURSING.**
By Dr. Stephenson.
Illustrated, 3/6 net, 3/10 post free.

**MODERN NURSING OF CONSUMPTION.**
By Dr. Jane Walker.
1/- net, 1/2 post free.

**MENTAL NURSING.**
By Dr. William Harding.
1/- post free.

**OUTLINES OF ROUTINE IN DISTRICT NURSING.**
By M. Loane.
1/6 net, 1/9 post free.

**A COMPLETE HANDBOOK OF MIDWIFERY.**
By J. K. Watson, M.D.
Profusely Illustrated, 6/- net, 6/4 post free.

**LECTURES ON THE NURSING OF INFECTIOUS DISEASES.**
By Dr. Woollacott.
2/6 net, 2/9 post free.

**NURSING IN DISEASES OF THE THROAT, NOSE AND EAR.**
By T. McLeod Yearsley, F.R.C.S.
2/6 post free.

**ELEMENTARY TREATISE ON THE LIGHT TREATMENT FOR NURSES.**
By Dr. Sequeira.
2/6 net, 2/9 post free.

**NURSING OF SICK CHILDREN.**
By Dr. Burnet.
1/- net, 1/1 post free.

**SIMPLE SANITATION:**
**The Practical Application of the Laws of Health to Small Dwellings.**
By M. Loane, Superintendent of District Nurses, Portsmouth.
Limp cloth, 1/- net, 1/2 post free.

**The Scientific Press, Ltd.,**

☞ *Complete Catalogue, post free, on application.*

**Publishers of Nursing Manuals, Case-Books, and Charts of all Descriptions.**

**28 & 29 SOUTHAMPTON STREET, STRAND, LONDON, W.C.**

